# Sex-Dependent Effects of Perinatal Inflammation on the Brain: Implication for Neuro-Psychiatric Disorders

**DOI:** 10.3390/ijms20092270

**Published:** 2019-05-08

**Authors:** Maryam Ardalan, Tetyana Chumak, Zinaida Vexler, Carina Mallard

**Affiliations:** 1 Department of Physiology, Institute of Neuroscience and Physiology, Sahlgrenska Academy, University of Gothenburg, 40530 Gothenburg, Sweden; maryam.ardalan@gu.se (M.A.); tetyana.chumak@gu.se (T.C.); 2 Department of Neurology, University of California–San Francisco, San Francisco, CA 94158, USA; zena.vexler@ucsf.edu

**Keywords:** neuroimmune, neuropsychiatry, perinatal, sex, microglia

## Abstract

Individuals born preterm have higher rates of neurodevelopmental disorders such as schizophrenia, autistic spectrum, and attention deficit/hyperactivity disorders. These conditions are often sexually dimorphic and with different developmental trajectories. The etiology is likely multifactorial, however, infections both during pregnancy and in childhood have emerged as important risk factors. The association between sex- and age-dependent vulnerability to neuropsychiatric disorders has been suggested to relate to immune activation in the brain, including complex interactions between sex hormones, brain transcriptome, activation of glia cells, and cytokine production. Here, we will review sex-dependent effects on brain development, including glia cells, both under normal physiological conditions and following perinatal inflammation. Emphasis will be given to sex-dependent effects on brain regions which play a role in neuropsychiatric disorders and inflammatory reactions that may underlie early-life programming of neurobehavioral disturbances later in life.

## 1. Introduction

The perinatal period is a vulnerable time in life. Preterm birth and long-term neurological sequelae such as cerebral palsy and cognitive problems have been associated with intrauterine infection/inflammation and neonatal sepsis [1,2]. Individuals born preterm have also higher rates of neurodevelopmental disorders such as schizophrenia, autistic spectrum disorder (ASD), and attention deficit/hyperactivity disorder (ADHD). These conditions are often sexually dimorphic and with different developmental trajectories. Both preclinical and clinical evidence suggests that many neuropsychiatric diseases originate prenatally or in early postnatal life. Animal models find strong connections between maternal exposure to infectious agents and altered brain and behavioral development in the offspring [3,4,5]. The timing of the immune activation during pregnancy appears to be an important factor, however, there are discrepant findings and vulnerability is likely multifactorial and influenced by other factors such as genetics, sex, and type of infection [6]. Further, it has been suggested that maternal infection causes epigenetic alterations leading to neurodevelopmental abnormalities in the offspring [7,8] and that epigenetic regulation differs in the male and female brain [9]. In many cases, sex predetermines the prevalence, age of onset, and progression of neurological and psychiatric conditions [10]. For instance, ASD, ADHD, and early onset schizophrenia have a male bias, while prevalence of depression and anxiety disorders is twice as high in females [11]. The association between sex- and age-dependent vulnerability to neuropsychiatric disorders has been suggested to relate to immune activation in the brain, including complex interactions between sex hormones, brain transcriptome, activation of glia cells, and cytokine production [12,13,14,15,16,17]. In this review, we will discuss sex-dependent effects on normal brain development and consequences of perinatal inflammation. Emphasis will be given to effects of sex on events in brain regions critical for neurodevelopmental and neuropsychiatric disorders. Further, we will focus on recent progress in understanding of how sex influences the inflammatory reactions that may underlie early-life programming of neurobehavioral disturbances later in life.

## 2. Sex-Dependent Effects on Physiological Brain Development

The central nervous system (CNS) is a highly heterogeneous organ with respect to structure, function and plasticity. Brain development is a sexually dimorphic process that in humans begins in the third gestational week with the differentiation of neural progenitor cells and continues through to late adolescence [18]. It was previously believed that sex-dependent differences in the brain are mainly related to brain functions in the context of reproduction. However, it has lately become clear that sex has an influence not only on areas regulating reproductive behavior, but also on general neurobiology and neurophysiology during brain development [19]. Already in utero, gonadal steroid hormones mediate sex differences on brain structure and function, including neurogenesis, synaptic pruning, dendritic branching, axonal growth, myelination, and apoptosis [20]. To acquire the male phenotype, the brain undergoes masculinization via androgen exposure, which peaks in the male rodent fetus at the end of gestation and falls quickly after the first postnatal day [21]. Exposure to sex hormones during brain development also modulates brain patterning and formation of neuronal networks. These alterations result in numerous permanent sex-related differences in the brain structure and function, which are reflected in behavior later in life [22]. 

The male brain has on average a higher total volume than the female brain, however, there are numerous anatomical and growth pattern differences between the sexes [23]. The amygdala, a brain region involved in modulation of emotions, learning, and memory, develops structurally faster in females and reaches its full growth potential approximately 1.5 years earlier than in males. At the same time, the final relative size of the male amygdala is larger than in the female. This is believed to be, at least partially, due to a longer period of development as a consequence of earlier pruning of neural connections in females [24,25] and higher number of androgen receptors and testosterone in the male brain [26]. Other brain areas are relatively larger in the female than male at birth, such as gray matter around the temporal-parietal junction (area involved in social behavior) [27]. The hippocampal volume increases faster in girls, particularly the left hippocampus [3]. In subcortical regions such as the thalamus, smaller volume has been observed in females irrespective of age [28]. Moreover, gray and white matter developmental trajectories are sexually dimorphic. Indeed, in a study including children and young adults, growth of cerebral white matter with age was linear but faster in females, while gray matter was nonlinear. Sex-by-age interactions showed larger cortical surface area in males up to age 15, while cortical surface area in females was relatively stable with increasing age [29]. 

In addition to the size of different brain regions, timing and magnitude of critical developmental processes in the brain like cell apoptosis and synaptic pruning differ in a sex-dependent manner [30,31]. Preclinical studies indicate that prostaglandin-dependent mechanisms are important as neuronal spine plasticity in the developing preoptic area (POA) is regulated by induction of prostaglandin-E2 (PGE2) synthesis through estradiol [32]. Indeed, cross-talk between calcium-dependent protein kinases, α-amino-3-hydroxy-5-methylisoxazole-4-propionic acid (AMPA)/kainate, metabotropic glutamate receptor signaling and PGE2 results in formation and stabilization of synapses [33] and leads to double spine density per unit of dendrites in males compared to females [34]. Since microglia is the main source of PGE2 in the brain, their number and morphological profile determine the level of PGE2 and indeed, higher microglia number accompanied with the higher production of PGE2 was found in the male compared to the female brain [16]. Thus, these data support the hypothesis that neuroimmune signaling is important for sex hormone effects on brain development and behavior [35], as well as modulation of neurotransmission [36].

It is not known how general these findings are as studies have mainly focused on the preoptic area, a region of the hypothalamus that is essential for sexual behavior. However, a higher rate of hippocampal neurogenesis in males compared to females indicates sex differences outside the context of reproduction [37]. Studies also demonstrate sex differences in dendritic morphology and synaptic patterning in hippocampal pyramidal neurons, which has been related to higher levels of suppressive miRNAs resulting in less proliferative effects of estradiol on the developing female hippocampus [36]. 

## 3. Effects of Sex on Glial Cell Development

The brain possesses its own immune cells mainly represented by glial cells, particularly microglia and astrocytes. Immune/inflammatory factors (e.g., cytokines, chemokines, and the complement system), hormones and growth factors link neuroimmune cells to other types of brain cells. The neuroimmune system is versatile and in addition to recognition of pathogens and clearance of debris in pathology, is critical for many functions during brain development. Indeed, astrocytes and microglia play important roles in the normal formation and plasticity of neuronal circuits during development and in processing information [38,39]. Microglia are important regulators of synapse function, plasticity, and circuit formation, and therefore, abnormalities in microglia morphology/function during early development may contribute to neurodevelopmental disorders such as autism, schizophrenia, and depression [40,41,42]. Similarly, astrocytes are critical for synapse formation and maturation [39] and also play a role in the pathogenesis of neurodevelopmental disorders [43]. Further, impairment of the brain’s excitatory/inhibitory balance through excess or lack of synaptic pruning by glia cells is believed to be linked to several neurodevelopmental disorders, including ASD and schizophrenia [44].

### 3.1. Microglia

Microglia make up approximately 5–15% of all cells in the human brain and apart from inflammatory functions, play a critical role in neurogenesis, apoptosis, synaptogenesis, synaptic pruning, and synaptic transmission [45]. Microglia differentiate from yolk-sac derived macrophages, which migrate to the brain during early embryonic (E) stages, E8.5–10 in cerebrum and E11 in cerebellum, and eventually account for the vast majority of the microglia population in the adult brain. Upon colonization, microglia proliferate throughout the embryonic and postnatal periods peaking in number on postnatal day (P)14 in rodents and then decrease to adult levels during the third postnatal week [46]. Based on the pattern of up- and down regulation of certain groups of genes, several phases in microglia development can be distinguished: progenitor (until E10.5), embryonic phase one (E12.5–E14.5), embryonic phase two (E16.5–P0), and adult stage [47,48]. Sex-dependent differences in gene expression pattern become noticeable as early as embryonic phase two, although they are not fully displayed morphologically at this time [48,49]. A recently introduced microglia-specific developmental index, based on global gene expression patterns, showed accelerated microglial development in female compared to male mouse brains [50]. In utero, environmental factors can affect microglia development in a sex-dependent manner, and for example, germ-free conditions of the mother were recently shown to impair microglia maturation to a higher extent in male compared to female fetuses [48]. 

During the embryonic period and at birth most murine microglia, in both males and females, exhibit a morphology of “activated” state, which is characterized by round amoeboid cells or cells with stout processes. With a few exceptions the number of microglia is comparable between sexes at this stage, however, at P4–5 the total number of microglia, as well as the number of cells with an activated morphology, is significantly lower in many regions of the female brain compared to the male brain [49,51]. These differences are believed to be due to an increase in the rate of testosterone-mediated-cellular proliferation in males rather than lower cellular survival in females [52].

In brain areas important for sexual differentiation, such as the POA, neonatal male mice have twice as many ameboid microglia as females, which is dependent on estradiol and PGE2 [16]. By P8, sex-dependent difference in microglial number in the hippocampus disappears due to a relative gain in microglia number in the female brain [51]. However, in many regions of the brain (parietal cortex, hippocampus, and amygdala) the number of activated microglia is higher in females compared to males at P30, reversing the sex dependent differences found at P4–5 and mirroring sex bias in vulnerability of males and females to specific neurodevelopmental disorders [49]. 

By the end of the third postnatal week, the predominant microglia phenotype in both sexes is characterized by long, thin processes (i.e., quiescent or so called “ramified”) [53]. As microglia during early brain development has been shown to have limited expression of steroid hormone receptors, it has been suggested that other neural cells that express steroid hormone receptors are responsible for the sex differences in microglia number and phenotype [54,55]. 

One of the crucial functions of microglia during development is phagocytosis of dead cells and inactive synapses as a part of synaptic pruning. At P3, female hippocampal microglia were found to possess higher number of cells with phagocytic cups and with higher expression of several phagocytic pathway genes compared to male cells [52,55]. Higher hippocampal expression of Iba-1 in P4 females compared to males may also testify to higher phagocytic activity of female microglia [56], as Iba-1 has been shown to be a key molecule in membrane ruffling and phagocytosis by macrophages/microglia [57]. Sex-dependent microglial phagocytotic activity may also be related to sex-dependent modes of neuronal apoptosis. For example, more profound Poly (ADP-ribose) polymerase (PARP)-1 mediated neuronal death in male pups, whereas more caspase-3 mediated neuronal death in female pups is apparent under ischemia-like conditions [58,59]. Between P8 to P15, there is a transient peak in phagocytic capacity of microglia in the hippocampus of both sexes, but with the peak shifted in time occurring earlier in female brain (closer to P8) than in male (closer to P15). In cerebellum, a part of the brain that matures relatively late compared to other brain regions, the number of microglia peaks at around P10, and by P17 immature microglia are almost fully replaced by microglia with high phagocytic activity. At the same age, male cerebellum has higher density of microglia with thin processes (more mature state) in the granular cell layer compared to females, but with no sex related differences in phagocytosis [60]. A recent human brain transcriptome study showed higher expression of genes associated with complement-dependent phagocytic function of microglia in males than females before birth, but downregulated in males versus females at postnatal stages [61]. However, at present, there is no direct evidence for the effect of sex on complement expression during brain development. 

Under physiological conditions, there is a sex-related bias in cytokine production by microglia and peripheral immune activation can result in different cytokine responses in male and female brains. Thus, microglia from P3 female mice have higher basic mRNA expression levels for pro-inflammatory cytokines TNF-α, IL-1β, IL-6, and anti-inflammatory cytokine IL-10 than males. However, in response to lipopolysaccharide (LPS) stimulation, male microglia tend to express more IL-1β mRNA than female-derived cells [16,55,62]. Further, Turano at al. showed greater potentiation of IL-6 production by male neonatal hippocampal microglia compared to female in response to LPS challenge [16,54]. Taken together, these findings suggest that sex differences in the morphology or number of microglia under physiological conditions are not necessarily followed by corresponding sex-dependent pattern in microglia function and response to stimuli. 

### 3.2. Astrocytes

Astrocytes are a subset of glial cells that develop from precursor cells in the ventricular and subventricular germinal zones and from radial glia [63]. In the mouse brain, astrocytes appear at around E18 and proliferate at least until P7 [64]. The major source of glia cells in the postnatal rodent cortex is the local proliferation of differentiated astrocytes [65]. Astrocytes are heterogenous cells both with regard to brain region and function and have been sub-classified into protoplasmic and fibrous astrocytes, located in the gray and white matter, respectively [66,67]. Expression of sex chromosomes in astrocytes and/or developmental exposure to sex hormones influence astrocyte development in males and females [68]. Astrocytes express steroid hormone receptors especially in brain regions that play a role in sexual differentiation and behavior [69,70,71]. Therefore, variation in the levels of estradiol has major effects on the maturation and morphology of astrocytes during development [72]. Prenatal hormone exposure leads to faster maturation of astrocytes in the male rat arcuate nucleus as evident by higher glial fibrillary acid protein (GFAP) immunoreactive surface area (including soma and cell branches) [73] and reduced maturation in the CA1 subfield of hippocampus in females as shown by less increase in GFAP content during pre-weaning. On the other hand, the size of astrocytes was larger in females than in males at P11 [74]. While no difference between sexes was demonstrated in immunostaining for the astrocytic intermediate filaments vimentin and GFAP at P7, at 4 weeks of age the surface density of vimentin was significantly lower and GFAP higher in males than in females [75].

Newborn females have higher rates of overall cell proliferation than males in the amygdala, which is a result of higher proliferation rates of astrocytes at P4 in females, an effect believed to be mediated through endocannabinoid receptors [76]. Another possible explanation for the higher cellular proliferation in female amygdala is that the lower number of microglia in this brain region leads to lower rate of phagocytosis of cells [77]. No differences in basal levels of cytokine production (IL-6, TNF-α, IL-1β, IL-10, and IP-10) have been described between male and female astrocytes [78,79]. 

In summary, sex-dimorphic effects on astrocyte proliferation and maturation during development is brain region and age dependent and environmental factors including sex hormones can affect astrocytes differently in males and females. 

### 3.3. Oligodendrocytes

Oligodendrocytes, the myelin-forming cells of the brain, develop from glial progenitor cells, so called oligodendrocyte precursor cells (OPCs). OPCs originate from several parts of the ventricular germinal zones of the embryonic neural tube and migrate throughout the developing CNS before differentiating into myelinating oligodendrocytes [80]. The sequence of events that characterize the transition from premyelinating to myelinating state has been well defined [81]. Sexual dimorphism is present in both young and old rodents with an increased density of oligodendrocytes of 20–40% in several brain regions in males compared with females, which is believed to at least partly depend on androgen exposure [82]. In vitro data show that progesterone can also directly stimulate oligodendrocyte precursors (NG2-, O4-, nestin- and platelet-derived growth factor α receptor-positive cells) and possibly accelerate their maturation into myelinating oligodendrocytes [83]. Progesterone effects on oligodendrocyte number are greater in females, which is due to changes in survival rather than proliferation. The apoptotic rate of oligodendrocytes in corpus callosum, as measured by active caspase-3, was 50% greater in females compared with males [84].

Accordingly, the causal relationship between sexual dimorphism of oligodendrocyte development needs to be considered in studies of neurodevelopmental disorders following perinatal insults.

## 4. Neuropsychiatric Disorders: Clinical Evidence of a Role for Infection/Inflammation

Significant evidence points towards a relationship between maternal infection/inflammation and neuropsychiatric disorders in the offspring. In many cases these outcomes are differentiated by sex, as outlined below and in Table 1.

### 4.1. Autism Spectrum Disorder (ASD)

The overall prevalence of ASD in US was recently reported to be 3.63% in boys and 1.25% in girls [100]. Although the risk of ASD diagnosis in children born preterm has decreased since the 1990s [101], meta-analyses demonstrate that low gestational age at birth remains a significant risk factor for ASD [102]. A cohort born extremely preterm showed a four time increase in ASD at 10 years of age, specifically in boys [103]. Similarly, in another study with early screening for signs of autism in a population of toddlers born preterm, male sex, history of chorioamnionitis, gestational age, and low birth weight were significant risk factors [104]. Low birth weight, specifically in girls, was associated with increased risk for autism accompanied by mental retardation [105]. ASD is typically diagnosed at 1–3 years of age. The early onset suggests that factors already before birth can be important in the pathogenesis of ASD and affect sex-dependent responses [106,107].

A large Finnish cohort study (*n* = 1.2 million) revealed that maternal C-reactive protein (CRP) was associated with increased risk of autism in children, although a significant interaction between maternal CRP and sex could not be demonstrated [87]. However, in another study, a relationship between repeated childhood infections and autism was observed to be stronger in girls compared with boys [88]. ASD has been associated with viral (rubella or cytomegalovirus) as well as bacterial and parasitic infections [107]. A large Danish nationwide register study (*n* = 1.6 million) investigated the correlation between maternal infection, based on the organism (viral and bacterial), organ, timing during pregnancy, and ASD. Although no specific microbe was identified, it was found that viral and bacterial infections during the first and second trimesters respectively and specifically respiratory infection correlated with increased risk of ASD in the offspring. Among viral infections, influenza was the most common and accounted for 25% of ASD cases [85]. In a recent clinical study, where 80% of patients with ASD were boys, more than half of the mothers had infection or antibiotic medication. Interestingly, infection itself was not associated with ASD, but maternal pharmaceutical treatment was significantly associated with ASD [86]. The authors suggested that either only severe infections requiring medication increased the risk of ASD or antibiotic therapy had detrimental effects on microbiome balance [108], although this was not directly tested. 

Clinical evidence indicates that imbalances in the composition of the microbiome may contribute to the development of ASD, as abnormal bacterial flora in the gastrointestinal (GI) tract has been demonstrated in autistic children [109]. More specifically, the level of Bacteroidetes was significantly higher in severely autistic children, while Firmicutes levels were significantly higher in the control group [110]. 

Alterations in the immune system and excessive expression of inflammatory mediators in the brain have been associated with the development of ASD [111]. Immune dysfunction and inflammation are intimately related, and it is therefore not surprising that levels of several major inflammatory cytokines and chemokines are markedly increased in autistic children, whereas levels of anti-inflammatory cytokines are decreased [112]. Children with ASD have, for example, increased IL-9 in peripheral blood mononuclear cells [113] and peripheral cytokine profiles at birth have been associated with severity of ASD in childhood [114]. Furthermore, plasma immunoglobulin levels are reduced in children with ASD [115], and the antibody response altered [116,117]. There are studies that also suggest a difference in the inflammatory profile in adult men and women with ASD. Men with Asperger’s syndrome (AS), a milder form of ASD, have increased levels of cytokines and pro-inflammatory molecules, while in female AS patients, growth factors and hormones such as androgens, growth hormone, and insulin-related molecules are important [118]. 

Inflammatory responses both in the mother and child have been associated with ASD. Elevated maternal serum cytokines (IFN- γ, IL-4, and IL-5) during mid-gestation were demonstrated in women giving birth to a child with autism [119]. Another study showed higher levels of TNF-β and IL-4 in amniotic fluid of female ASD cases, but IL-5 in amniotic fluid of male ASD patients. Authors suggested two possible explanations for these results: either the immune system in girls was more “ready” to react with higher cytokine production in response to infection compared to boys, or different brain regions are involved in the pathophysiology of autism between the sexes [120]. Reduced risk of ASD in children from mothers with a history of antipyretic medication with mild anti-inflammatory effects, such as acetaminophen, has been reported [121,122]. In contrast, a recent study showed that maternal usage of anti-pyretic treatment was related to higher risk of ASD, specifically in males [123].

Human studies implicate peripheral monocytes as major contributors to the immunological component of ASD [112], which in part occurs through Toll-like receptor (TLR) activation [124,125]. An altered monocyte response to activation of several TLRs has been observed in children with ASD [126,127]. The role of TLRs is age-dependent, and based on particular heterodimers formed by TLR1/2/3/4, TLRs elicit context- and tissue dependent effects, from injurious to beneficial [128]. However, it is clear that systemic TLR stimulation can cause developmental delays [129]. Speculatively, cell dysfunction in response to TLR activation could lead to adverse neuroimmune interactions, long-term immune changes, and the pathophysiology observed in ASD.

### 4.2. Attention-Deficit/Hyperactivity Disorder (ADHD)

ADHD is a neurodevelopmental disorder with a prevalence of 5.3% in children and 2.4% in adults, with predominance in males [130,131,132]. In a series of studies by the ELGAN investigators, numerous risk factors of ADHD in 10-year old children were identified, including immaturity at birth and inflammation [133]. In addition, a positive correlation between systemic inflammation during the first postnatal month among children born extremely preterm and risk of ADHD regardless of sex was observed [134]. It has been hypothesized that ADHD is the result of activation of neuroinflammatory pathways in the fetus in response to allergy or autoimmune disease in the mother [89]. A population-based case-control study indicated that several maternal somatic diseases with immune components such as multiple sclerosis, rheumatoid arthritis, and asthma were associated with increased risk of ADHD in offspring of both sexes. Interestingly, in addition, paternal immune activation was related to ADHD, but risk estimates were lower than for maternal chronic immune disease [135]. Increased levels of antibodies against the dopamine transporter have also been observed in ADHD patients, giving further support to a role of the immune system in this disorder [136]. 

### 4.3. Schizophrenia

In Europe, the incidence of schizophrenia is 15.2 per 100,000 persons and the morbidity risk of schizophrenia is 7.2 per 1000 persons [137,138]. In general, the age of schizophrenia diagnosis is different between males and females with a higher frequency of diagnosis in boys and young men, while diagnosis in women is more common from middle age and onwards. 

Several studies have reported associations between maternal infection and increased risk of schizophrenia in the offspring. Influenza A virus infection during the first trimester of pregnancy was associated with sevenfold increased risk of schizophrenia in the child [139]. Also, maternal exposure to toxoplasma gondii and cytomegalovirus increased the risk of schizophrenia in the offspring [140]. During years with high incidence of influenza, an increased rate of schizophrenia was observed in females, but not in males [141]. In addition, prenatal exposure to bacterial (specifically respiratory) infections in the first trimester of pregnancy were associated with increased risk of schizophrenia [96]. It has been hypothesized that maternal antibodies to infectious agents can pass the placenta and cross-react with molecules important during the development of the nervous system [142]. 

A case-control study observed higher prenatal IL-6 levels among male schizophrenics compared to male controls, while TNF-α was lower among female schizophrenics than female controls. Further, the study indicated an important interaction between immunologic processes and offspring sex in differential risk for psychoses [98]. These studies suggest sex-dependent mechanisms in the development of schizophrenia, which may affect therapeutic strategies. For example, treatment of schizophrenia patients with anti-psychotic drugs showed different responses between male and female with sex-specific changes in the levels of IL-13, and macrophage derived chemokines [143].

### 4.4. Depression

The lifetime prevalence for major depressive disorder (MDD) was recently reported to be over 28% [144]. MDD is considered to be a strongly sex biased neuropathology, with women being diagnosed twice as often as men. 

Birth cohort studies have identified prenatal conditions, including maternal immune activation, as significant risk factors for MDD indicating the importance of the prenatal period as a sensitive period for the establishment of lifelong risk for depression [3]. One study showed that women exposed to type A2/Singapore influenza during the second trimester of pregnancy gave birth to children at increased risk for depression in adulthood. This effect was markedly stronger in men versus women (16% vs. 2%) [95]. Proinflammatory cytokine release following both bacterial and viral infections has been associated with a range of depressive symptoms [145,146]. A recent clinical study investigated the association between biomarkers of maternal immune activity in serum during the second half of pregnancy and the risk of MDD in adult offspring and found that increased risk of depression after exposure to higher prenatal TNF-α: IL-10 levels was restricted to male offspring [91]. Thus, current clinical data does not provide consistent evidence to explain perinatal inflammation as an important etiological factor for depression in women, but there is some support in men.

In summary, maternal infection during pregnancy and infant/childhood infections increase the risk of several neurodevelopmental disorders, suggesting that inflammatory responses are important in the pathogenesis. However, few studies demonstrate a clear sex-dependent link between infection and neuropsychiatric outcome. Thus, sexually dimorphic functional disorders in the human are supported mainly by prevalence and the pathogenesis or pathophysiology of each disorder remains to be confirmed in clinical studies.

## 5. Animal Models of Perinatal Inflammation: Behavioral Abnormalities Related to Neurodevelopmental Disorders

Significant evidence suggests an interactive effect of fetal sex and perinatal neuroinflammation in the pathogenesis of neurodevelopmental disorders, where factors such as the dose of the immunogen, the gestational timing, and acute versus chronic nature of the immune challenge are important [147]. An array of rodent models have been developed to explore the link between altered neural-immune interactions and ASD and/or other neurodevelopmental disorders. Considering data in humans on the role of innate immune receptors, TLR activation is frequently used to trigger the immune response in preclinical studies (Table 2). In such models, synthetic ligands to individual sub-types of TLRs are often used, including polyinosinic-polycytidylic acid (Poly I:C, a TLR3 ligand) and LPS (a TLR4 ligand).

Studies of maternal exposure to the viral mimetic poly I:C have consistently showed modified behavior linked to neuropsychiatric disorders [169,170,171]. For example, male offspring from poly I:C injected mothers display decreased preference for the social chamber and extremely high repetitive behavior [163,172], which was suggested to occur at least in part via sex-dependent innate immune mechanisms [173]. Similarly, schizophrenia-related behavioral abnormalities, such as deficits in prepulse inhibition (sensorimotor gating) and latent inhibition effect of associative learning, were reported in the offspring of poly I:C treated mothers, particularly when inflammation was induced early in pregnancy [174,175]. On the other hand, prenatal poly I:C exposure on G17 resulted in deficits similar to negative symptoms in schizophrenia such as reduced social interaction, anhedonia, and alterations in locomotor and stereotyped behavior in both male and female offspring [161], while enhanced conditioned fear behavior was only observed in female animals [176]. In addition, male offspring, but not female, displayed cognitive symptoms as shown by alterations in executive function [158]. 

Prolonged prenatal LPS exposure during the last two thirds of gestation resulted in deficits in sensorimotor gating and impairment in social interactions and exploration more in adult male than in female rats [153] and LPS in late gestation (G15, 16, and 17) increased anxiety- and depression-like behaviors in male offspring [177]. Depression-like symptoms were reflected in increased immobility time (forced swim test) in male and female rats prenatally exposed to LPS [178]. Further, intraperitoneal LPS administration on P4 and P5 disrupted avoidance learning in adult male, but not female subjects [179]. 

A recent study also indicates that prenatal TLR7 activation results in sex-dependent neurobehavioral abnormalities and specific sex-dependent gene regulation in the dorsal striatum [180]. In addition to studies using specific TLR ligands, *Group B Streptococcus* (GBS) induced maternal infection during pregnancy in rats has been linked to increased spontaneous locomotor activity and decreased inhibition, particularly in female offspring [181].

A few studies in larger animals, such as ovine or rhesus monkey, have examined the effect of maternal immune activation on brain development in connection with ASD and schizophrenia [167,171,182,183,184]. Most of these investigations did not consider sex-dependent effects. However, one study in the rhesus monkey examined the pathogenic potential of immunoglobulin G (IgG) in the first and second trimesters of pregnancy on ASD and found that male IgG-ASD offspring had enlarged brain volume compared with controls, particularly in the white matter of the frontal lobes [167]. To our knowledge, behavioral studies have not been performed in large animal models. 

## 6. Pre-Clinical Evidence of Pathogenesis in Neurodevelopmental Disorders

The underlying mechanims of the altered behavior following perinatal inflammation are not fully understood, but likely include a combination of genetic and neurobiological factors, such as neurotransmitter release, neurite outgrowth, and axon guidance [185,186]. For example, interaction between mutant human disrupted-in-schizophrenia 1 (*mhDISC1*) and maternal immune activation has been implicated in schizophrenia and mood disorders [187] and sex-dependent difference in development of hypothalamic and arousal circuitry seems to be a key factor in determining male and female risk of schizophrenia [188,189]. Data support, at least partially, that sex-specific differences in cognitive inflexibility following maternal poly I:C correlate with neurotransmitter levels including reduced dopamine, glutamate, γ-aminobutyric acid (GABA) and glycine contents in the medial prefrontal cortex and hippocampus [161]. Moreover, epigenetic remodeling of GABA synthesis was linked to abnormal synaptic plasticity followed by impairment of working memory and social interaction [190]. LPS-induced behavioral changes have also been associated with sexually dimorphic effects on GABAergic interneurons, with decreased total number of parvalbumin and GAD67-positive neurons in the medial prefrontal cortices of adult females, whereas similar cellular changes were detected in the hippocampus of males. It has also been shown that antenatal poly I:C exposure alters serotonin synthesis within the placenta, which affects serotonin-dependent neurogenic processes during fetal neurodevelopment and may have implications for neurodevelopmental disorders [191].

Gastrointestinal dysfunction is common in autistic patients. Recently, susceptibility to neurodevelopmental diseases in the offspring following maternal immune activation was associated with maternal microbiota and IL-17α signaling during gestation [108]. In support, prenatal and postnatal treatment with a by-product of enteric bacteria, propionic acid, resulted in autistic-like behavior in female rats [192]. The inbred mouse strain *BTBR T+ tf/J* (BTBR) exhibit deficits that mimic core behavioral changes in autism [193] and interestingly, sex-related alterations of gut microbiota composition were shown in the BTBR ASD mouse model [194]. This mouse model has also provided evidence for a causal relationship between peripheral immune phenotype and social behavior by demonstrating that bone marrow transplant from control mice increases sociability in BTBR ASD mice [195]. 

## 7. Sex-Dependent Effects of Perinatal Inflammation on Glial Cells and Brain Inflammation

### 7.1. Microglia Activation

Maternal immune activation was shown to induce microglial activation and upregulation of pro-inflammatory genes, particularly impacting on a shift towards a more advanced developmental phenotype by upregulation of the transcription factor MAFB, an important component of adult microglia programming, although sex-dependent effects were not been determined [196]. Transient depletion of microglia during the neonatal period using liposomal clodronate affected early life programming and subsequent juvenile and adult motivation behavior, but no sex-dependent differences were observed except that females showed attenuated corticosterone response after acute stress in adulthood [197]. Mice lacking microglial *atg7*, a protein essential for microglia autophagy, displayed impaired sociability but normal social recognition in both male and females [198]. Furthermore, microglia abnormality can lead to depression-like symptoms and some forms of depression are considered a microglial disease (microgliopathy) [41].

Estradiol, the major female sex steroid, was shown to modulate LPS-induced inflammation in cultured microglia in a sex-depend manner [199]. Microglia derived from male neonatal mice expressed higher levels of mRNA for IL-1β than females following LPS stimulation. On the other hand, estradiol had anti-inflammatory effects on male microglia, while pro-inflammatory effects were observed in female microglia [199]. An investigation to link steroid hormones and brain colonization of microglia demonstrated that neonatal exposure to ethinyl estradiol or Bisphenol A (a mimic of sex steroid hormone exposure) doubled the total number of microglia in the dentate gyrus of hippocampus in males by P12 by increasing the number of intermediate microglia, but without affecting the number of ramified microglia [200,201]. However, elevated juvenile anxiety was observed in both females and males in this model [200], and therefore, no clear conclusions of a link between neuroinflammation, sex and dysfunctional behavior can be drawn. 

Evidence for a role of microglia pruning in neuropsychiatric-like behavior comes from studies in mice lacking the chemokine receptor *Cx3cr1*. These mice exhibit a transient reduction of microglia and synaptic pruning during the early postnatal period, which leads to transient increase in spine density, decreased functional brain connectivity, deficits in social interaction, and increased repetitive-behavior phenotypes [202]. In a model of maternal immune activation, which results in ASD-like behaviour, reduced hippocampal expression of *Cx3cr1* and increased spine density was observed in male offspring [152]. Rett syndrome is an X-linked ASD characterized in most cases by mutation of the *Mecp2* gene and mice with *Mecp2* gene deficiency develop symptoms similar to those seen in people with Rett syndrome [203]. The disease symptoms were related to microglia phagocytic activity and it was shown that transplantation of wild type bone marrow into the *Mecp2*-null host stopped disease progression. Interestingly, targeting glia cells with anti-inflammatory treatment (N-acetyl cysteine) improved behavioral outcomes in *Mecp2*-null mice [204]. 

Activation of the tryptophan-kynurenine pathway in microglia by immune challenges has been suggested to participate in the pathogenesis of depressive-like behaviors [205]. The kynurenine pathway and its potent stimulation by proinflammatory cytokines, particularly by interferon (IFN)-γ, indicates a role for the neuroimmune system in regulation of various neurotransmitters [206]. In the brain, the cytokine inducible enzyme indoleamine 2,3-dioxygenase (IDO) is involved in tryptophan degradation. Therefore, changes in IDO activity following inflammation might result in changes in serotonin levels secondary to tryptophan metabolism. Inhibition of the tryptophan-kynurenine pathway during gestation in rat alters synaptic plasticity in the hippocampus of the offspring [207]. There are, however, no direct studies investigating inhibition of the tryptophan-kynurenine pathway on behavior and sex-dependent effects. 

Taken together, microglial activation occurs following both maternal and neonatal immune activation. However, with few exceptions there are no consistent data to show sex-dependent effects and little evidence that directly links microglia activity and subsequent behavior in animals. Treatment by agents with specific inhibitory effects on microglia may help to better understand details about sex-dependent effects of early life immune activation on later-life behavioral impairment [62,197]. For instance, mutation of the microglia activity-dependent neuroprotective protein (ADNP) has been observed in ASD patients, especially in male patients with more severe intellectual disability [208]. In support, a preclinical study demonstrated that ADNP mutation resulted in severe cognitive impairment in male mice compared with female mice [209]. 

### 7.2. Astrocyte Activation

Clinical studies suggest that the serum level of brain-derived neurotrophic factor (BDNF) is an important biomarker in autistic individuals [30]. Astrocytes are one of the sources of BDNF, a member of the neurotrophin growth factor family with an important role in synaptogenesis in brain development and even adulthood [210]. However, a significant increase in the level of hippocampal BDNF was observed in both males and females following neonatal LPS, thus BDNF is unlikely to explain sex-dependent effects in astrocytes [202]. 

Sex-dependent activation of astrocytes in response to adverse environmental factors has been observed in several studies. Female astrocytes sustain greater cell death following exposure to inflammatory mediators (LPS, IL-1β, or TNF-α) in combination with oxygen–glucose deprivation. Therefore, it has been suggested that female astrocytes have the ability to change into an activated state more readily and thus be more sensitive to cell-death signals compared to male astrocytes [211]. 

Astrocytes are the most active steroidogenic cells in the brain and sex-dependent responses in steroidogenic regulator proteins such as TSPO (translocator protein) have been observed in response to LPS [212]. It was shown that at the basal level there was no significant difference in the mRNA levels of IL-6, IP-10, TNF-α, and IL-1β between male and female cortical astrocyte cultures, however, LPS induced higher mRNA levels of IL-6, TNF-α, and IL-1β in astrocytes derived from males compared to females. Furthermore, it was shown that astrocytes cultured from androgenized female mice responded to LPS similarly to male astrocytes by increasing mRNA expression of IL-6, TNF-α, and IL-1β to comparable levels, suggesting that perinatal testosterone programs astrocyte responses to pathological stimulation [78]. Chistyakov et al. confirmed a sex-biased response of astrocytes to LPS represented by different levels of cytokine and arachidonic acid metabolite production by male and female astrocytes [79]. 

Another possible explanation for a differentiated response may relate to the higher expression of TSPO in female astrocytes exposed to LPS, as it is known to regulate reactive gliosis and apoptosis [213,214]. Interestingly, after LPS treatment, female astrocytes produced more IL-10, COX-2, 6-keto-PGF1a, and thromboxane (TX) B2, and less TNF-α, PGE2, and PGD2, compared with male astrocytes. Further, co-treatment with Trilostan, an inhibitor of 3-Hydroxysteroid dehydrogenase, inhibited the LPS-induced production and release of PGE2, PGD2, and 6-keto-PGF1a in female astrocytes, while it increased expression of TNF-α, TXB2, and 6-keto-PGF1α in male astrocytes [79]. 

Thus, these studies show that not only do male and female astrocytes respond differentially to inflammatory challenges but can also react in dissimilar manner to interventions. Sex-dependent hormonal modification of immune responses in astrocytes is very complex and requires further investigation.

### 7.3. Effects on Oligodendrocytes

Several studies have indicated involvement of oligodendrocytes in the pathophysiology of neuropsychiatric disorders, such as myelination deficits in various brain regions in schizophrenia [215,216,217]. In infants born preterm, white matter injury and myelin deficits are increasingly being recognized as risk factors for ASD and other neuropsychiatric disturbances [218]. Maternal immune activation at E9.5 is linked to generation of Olig2-positive cells in the medial ganglionic eminence of the fetal murine telencephalon [219] and it has been suggested that deficits in myelination and axonal abnormalities following maternal immune activation could be underlying schizophrenia-related behaviors seen in adulthood [220]. Sex-dependent effects on oligodendrocytes following maternal inflammation point towards more significant alterations in males. Maternal inflammation induced by GBS infection resulted in white matter injury, oligodendrocyte loss, and shrinkage of periventricular external capsules thickness, alterations which were associated with autistic-like behavior only in male rat offspring [221]. It was suggested that these neuropathological changes were related to a deficit in oligodendrocyte maturation rather than cell death *per se*. Interestingly, the distribution of white matter injury appeared to differ depending on which inflammatory stimuli was used. White matter injury following GBS was limited to the periventricular white matter underlying mid-frontal and parietal lobes and was not associated with gliosis [221], while brain injury following LPS injection included both external and internal capsule injuries, which were associated with gliosis [222,223]. However, no sex-dependent differences in the pattern of white matter injury following maternal inflammation in connection with neurodevelopmental disorder have been reported.

### 7.4. Inflammatory Responses

Animal models of maternal infection have shown that dysregulation of cytokines can result in behavioral abnormalities in the offspring [224]. However, relatively little is known about the effect of sex on inflammatory responses and the relation to neurodevelopmental disorders. Osborne et al. showed increased hippocampal expression of TNF-α, IL-1β, and CD11b in both sexes at 8 h after *E. coli* infection in P4 mice. At the same time, decreased expression of BDNF was found only in males, while elevated IL-6 expression was only observed in the female cerebellum. Levels of TNF-α and CD11b normalized by 24 h after initiation of the infection, while elevation of IL-1β persisted. Interestingly, these differences were not reflected in the morphology of microglia following neonatal infection with *E. coli*. Moreover, no significant difference in the expression of inflammatory cytokines and chemokines between males and females in the spleen and serum was observed [56]. Given the important role of IL-1β in learning and memory processes in the hippocampus [225], the long-lasting increase in IL-1β expression in male rats may give an explanation for the potentiation of deficits in learning and memory later in life [226]. 

In the cerebellum of children with autism, a significant increase in IL-6 expression has been demonstrated [227]. IL-6 has been shown to have long-term effects on offspring brain development and behavior following maternal immune stimulation [228] and studies suggest that IL-6 is as a key intermediary molecule that predisposes to schizophrenia and autism [229]. In primary cortical neuronal cultures from rats, IL-6-induction significantly reduced the complexity of neuronal dendritic arborization, an effect that was linked to risk for schizophrenia [230]. Another study showed that prenatal treatment with IL-6 resulted in neuronal loss mainly in the hippocampal CA2 and CA3 areas of male offspring while in the hippocampal CA1 subregion of female offspring [231]. On the contrary, another study in mice observed decreased levels of IL-6 and increased IL-4 in both male and female mice following neonatal LPS administration [165]. At P35 and P70, male mice showed increased depressive-, anxiety-like, and repetitive behaviors accompanied with working memory deficits, which were not related to specific cytokine changes but to a general marker of inflammatory response (myeloperoxidase), hippocampal nitrite levels and reduced parvalbumin (a marker for GABAergic interneurons) expression. Interestingly, lower parvalbumin protein expression was specifically observed in the hippocampus of males exposed to LPS and resulted in disruption in the balance of excitation/inhibition and social behavior abnormality [165].

In addition to the direct effects of maternal infection, maternal stress-induced activation of immune pathways also induces neurobehavioral abnormality later in life. Maternal stress induces placental inflammation with increased levels of the proinflammatory cytokines IL-6 and IL-1β, specifically in male placentas, which is followed by locomotor hyperactivity, a hallmark of dopaminergic dysregulation [162]. Importantly, effects of nonsteroidal anti-inflammatory drug treatment (NSAIDs) on behavior in adulthood is different between male and female by showing normalized IL-6 levels in male tissue only [162].

Inappropriate complement activation during synapse development can alter neural connectivity by excessively targeting synapses for elimination [232]. In normal developing brain, complement C system proteins are synthesized locally by resident neurons, microglia, and astrocytes [233] and activates and promotes microglia phagocytosis resulting in further synaptic pruning [234]. The expression of C3 is lower in males versus females [235]. Neonatal treatment with C3a complement-derived peptide improves cognitive function in mice after injury, however, no sex differences were reported [236,237]. It has also been indicated that abnormal complement cascade activation may play a role in autism and psychiatric disorders such as schizophrenia [238,239]. For instance, a recent clinical study demonstrated that human C4 protein is localized to neuronal synapses, dendrites, axons, and cell bodies, and that there are alterations in C4A and C4B expression and excessive complement activity in the brains of schizophrenic patients [240]. A preclinical study also showed that in a *C1q* knockout animal model, the number of neuronal dendrites and spine density in sensorimotor cortex was higher, suggesting that C1q-dysfunction may induce ASD due to the failure of elimination of excessive excitatory synapses [241]. In contrast, increased C1q expression in serum from ASD children may be interpreted as a compensatory elevation to defects in C1q function [242]. Additionally, inhibition of C5a receptors during embryogenesis leads to abnormal brain development and behavioral deficits [243]. To our knowledge, there are currently no studies reporting on sex-dependent effects of the activation of the complement system after perinatal inflammation and subsequent behavior.

## 8. Conclusions

This review on sex-dependent effects of perinatal inflammation on the brain, with special implications for neuro-psychiatric disorders, has focused on rodent and human studies, as there are few investigations using large animals. The studies indicate that perinatal immune activation increases the risk of neuropsychiatric disorders overall, however, sex-dependent effects are often unclear. The interactions between perinatal inflammation, sex, and behavior in offspring are clearly complex and interpretation of the research is further complicated by use of different species, different immune agents, different ages, and timing of exposure. Thus, there is no consistent data on sex-dependent hyperactive or anxiety-related behaviors in offspring following pregnancy-related inflammation. Similarly, results on sex-dependent schizophrenia-related and autistic-like behaviors following inflammation are mostly inconclusive, except for several studies indicating impaired cognitive and executive function and social interaction impairment specifically in male offspring. 

The field is, however, evolving both conceptually and methodologically. Recently a “three-hit” (genetic load × environmental factor × sex) theory of pre-disposure to ASD [149] was introduced that may move the field forward conceptually. In this study, a contactin-associated protein-like 2 (*Cntnap2*) mutant mouse model of autism was used. The study showed vulnerability of males following maternal LPS-administration with respect to social recognition and histone modifications, which was associated with down-regulation of the *Crhr1* gene expression in the hippocampus [149]. Moreover, dysregulation of immune-related genes has been shown in the brains of patients with neuropsychiatric disorders such as schizophrenia [244], raising the possibility that immune-induced inflammation increases vulnerability of the brain that already is at risk due to genetic influences [245,246]. These studies also imply epigenetic modifications as important mediators of interactions between genes and environment. Thus, environmental factors, such as perinatal inflammation, during critical periods of brain development may cause imprinting of inflammatory genes that underlie early-life programming of neurobehavioral disturbances later in life [247]. Future studies are warranted to answer questions regarding the role of specific neuroimmune elements following perinatal inflammation that in a sex-dependent manner impact on neuropsychiatric disorders.

## Figures and Tables

**Table 1:** Clinical studies on perinatal immune activation and neuropsychiatric outcome.

Immune Activation	Neuropsychiatric Outcome
Maternal bacterial and viral infections [85]	Relationship between maternal infection and autism without specification on sex effect
Maternal and infant infections [86]	Relationship between maternal, infant infection and autism without specification on sex effect
Maternal early gestational C-reactive protein (CRP) [87]	Relation between maternal C-reactive protein (CRP) and risk of autism in both sexes
Children hospitalized for infection [88]	Stronger association of ASDs/infantile autism for girls compared with boys
Maternal autoimmune disorders [89]	No sex difference in ADHD prevalence
Maternal stress-infection interactions during pregnancy [90]	Association of maternal infection during the second trimester with depressive symptoms in adulthood for both sexes
Maternal cytokine levels [91]	Association TNF-α to IL-10 ratio in maternal serum with depression in a sex-dependent manner
Maternal herpes simplex virus 2 IgG level [92]	Association with schizophrenia without specification for sex effect
Maternal influenza infection [93]	Association with schizophrenia without specification for sex effect
Maternal influenza infection [94]	Association with schizophrenia without specification for sex effect
Maternal influenza infection [95]	Association with major depressive disorder without specification for sex effect
Maternal bacterial infection [96]	First-trimester infection, upper respiratory tract and gonococcal infections were associated with elevated risk of schizophrenia without specification for sex effect
Maternal influenza infection [97]	Association with schizophrenia without specification for sex effect
Maternal serum level of cytokines [98]	Schizophrenic males with high IL-6 levels and lower TNF-α levels among schizophrenic females
Level of cytokines in plasma samples from children diagnosed with ASD [99]	Negative correlation of ASD symptom severity with levels of IL-1β, IL-8, MIP-1β, and VEGF in females, but not in males

**Table 2:** Preclinical studies of perinatal immune activation with neuropsychiatric-like outcome.

Genetic Background	Immune Activation	Behavioural Outcome	Neuropathological Outcome
Sprague Dawley rats [148]	Maternal LPS (*Escherichia coli*, 026:B6, 0.1 mg/kg) at E11	Autistic-like behavior in male	Decreased glutamate, increased corticosterone levels within the hippocampus in males, Decreased levels of NR2B protein in both male and female
Cntnap2 KO and WT mice [149]	Maternal LPS (0.3 mg/kg) at E7	Autistic-like behavior in male	Lower corticotropin-releasing hormone receptor-1 (Crhr1) gene expression in the left hippocampus of male mice
C57BL/6J mice [150]	Maternal LPS (*Escherichia coli*, O127:B8, 0.075 mg/kg) at both E11.5 and E12 or Poly I:C (20 mg/kg) at E12.5	Autistic-like behavior (from both LPS and Poly I:C-treated mothers)	Not applied
C57/BL6 and BTBR mice [151]	Maternal Poly I:C (20 mg/kg) at E12.5	Autistic-like behavior only in BTBR males	Not applied
C57/BL6 mice [152]	Maternal LPS (*Escherichia coli*, 0111:B4, 0.1 mg/kg) at E15	Autistic-like behavior in male and female	Increased number of spines, reduced expression of the fractalkine microglial receptor (CX3CR1) in the hippocampus of only male
Wistar rats [153]	Maternal LPS (*Escherichia coli*, 026:B6, 1 mg/kg) every other day from E7 until birth	Schizophrenia-related behavioral phenotypes in both male and female	Decrease in the total number of PV- and GAD67-positive neurons in the medial prefrontal cortices of adult females, but only in the hippocampus of adult males
Wistar rats [154]	Maternal LPS *(Escherichia coli*, 026:B6, 1 mg/kg) every second day of pregnancy from E7 until birth	Schizophrenia-related behavioral phenotypes in both male and female	Elevated plasma level of corticosterone and a decrease in the glucocorticoid receptor level in the hippocampus and the glucocorticoid receptor-associated proteins FKBP51 concentration in the frontal cortex in both male and female
Swiss-Webster mice [155]	Maternal LPS (*Escherichia coli*, 0111:B4, 0.2 mg/kg) or Poly I:C (20 mg/kg) at E9	Not applied	At PD28, increased GAD67 expression in the ventral stratum oriens in male mice prenatally treated with LPS, and in female mice prenatally treated with poly I:C
Wistar rats [156]	Maternal LPS (*Escherichia coli*, 0111:B4, 0.1 mg/kg) at E15 and E16	Schizophrenia-related behavioral phenotypes in both male and female rats	Female rats showed weaker cognitive impairments, less demyelination and only a modest loss of PV expressing cells
Long-Evans rats [157]	Maternal Poly I:C (4 mg/kg) at E15	Schizophrenia-related behavioral phenotypes (Male displayed significantly impaired object-in-place recognition memory; disturbed prepulse inhibition (PPI) in both sexes)	Not applied
Long-Evans rats [158]	Maternal Poly I:C (4 mg/kg) at E15	Schizophrenia-related executive function that involve the prefrontal cortex (PFC) in male rats	Not applied
Sprague Dawley rats [159]	Neonatal LPS (*Escherichia coli*, 026:B6, 0.1 mg/kg) at P14	No anhedonia after both, neonatal and 2-hit LPS challenges	Elevated expression of hypothalamic COX-2 in male after neonatal LPS
Sprague Dawley rats [160]	Maternal LPS (*Escherichia coli*, 055:B5, 0.5 mg/kg) at E19	Impaired spatial recognition in males	Increased protein carbonylation, decreased a-Tocopherol and reduced/oxidized glutathione ratio, impaired NMDA synaptic currents and LTP in male hippocampus
C57BL/6 mice [161]	Maternal Poly I:C (5 mg/kg) at E17	Schizophrenia-related behavioral phenotypes in both male and female mice	Decreased dopamine level in prefrontal cortex and ventral hippocampus in both male and female Male: reduced glutamate, aspartate, taurine in prefrontal cortex Female: decreased g-aminobutyric acid, taurine in dorsal hippocampus, increased 5-hydroxytryptamine levels in amygdala and nucleus accumbens
C57/Bl6:129 [162]	Maternal stress (E1–E7)	Stress induced locomotor hyperactivity in males (ADHD)	Both sexes: increased proapoptotic factor, Fas ligand in placenta Male: increased placental cytokines, decreased D2 receptor in nucleus accumbens, increased D1 receptor in prefrontal cortex Female: decreased CCL2, dopamin receptor levels were not analyzed
Wistar rats [163]	Maternal LPS (*Escherichia coli*, 026:B6, 0.1 mg/kg) at E15	Reduced frequency of juvenile play (social) behavior in males	Reduced arginine vasopressin mRNA expression in the medial amygdala and bed nucleus of the stria terminalis only in males
Wistar rats [164]	Maternal LPS (*Escherichia coli*, 026:B6, 1 mg/kg LPS) on alternate days during pregnancy	Schizophrenia-related behavioral phenotypes in both male mice	Astroglial and microglial reactivity in different brain regions only in male Increased blood levels of IL-6, IL-2 in male and female
Swiss mice [165]	Neonatal LPS (*Escherichia coli*, 055:B5, 0.05 mg/kg) at P5 and P7	Depressive like, risk-taking, anxiety-like, and repetitive behaviors with working memory deficits in male Prepulse inhibition (PPI) deficits in female	Increased levels of IL-4 (PFC, hippocampus) and decreased levels of IL-6 (PFC, hippocampus) and increased BDNF levels in both male and female
Wistar rats [166]	Maternal Poly I:C (4 mg/kg) at E15	Not applied	Reduced hippocampal size in both male and female
Rhesus monkeys [167]	Maternal purified IgG, at E30, 44, 58, 72, 86, 100	Autistic-Like Behavior in male and female	Significant higher total brain volume in the age 3 and 6 months of life only in male IgG-ASD^37/73 kDa^ especially in frontal and occipital lobes
C57BL/6 mice [168]	Neonatal Poly I:C (10 mg/kg) at P8	Not applied	Activation of caspase-dependent pathways is earlier in female mice than male mice Higher GFAP expression 14 h after injection only in male mice Persistence of microglial activation at 7 days after poly I:C only in female mice

## Abbreviations

+	Positive
ADHD	Attention deficit/hyperactivity disorder
ADNP	Activity-dependent neuroprotective protein
AMPA	Α-amino-3-hydroxy-5-methyl-4-isoxazolepropionic acid
AS	Asperger’s syndrome
ASD	Autistic spectrum disorder
Atg	Autophagy related
BDNF	Brain-derived neurotrophic factor
BTBR	BTBR T+ tf/J mouse strain
CA	Cornu ammonis
CNS	Central nervous system
Cntnap2	Contactin-associated protein-like 2
COX	Cyclooxygenase enzyme
CRP	C-reactive protein
Cx3cr1	C-x3-c motif chemokine receptor 1
E	Embryonic day
*E. coli.*	*Escherichia coli*
GABA	γ-aminobutyric acid
GAD	Glutamic acid decarboxylase
GBS	Group B streptococcus
GFAP	Glial fibrillary acidic protein
Iba-1	Ionized calcium binding adaptor molecule 1
IDO	Indoleamine 2,3-dioxygenase
IFN	Interferon
IgG	Immunoglobulin G
IL	Interleukin
IP	Interferon gamma-induced protein
LPS	Lipopolysaccharide
MAFB	V-maf avian musculoaponeurotic fibrosarcoma oncogene homolog B
MDD	Major depressive disorder
MECP2	Methyl CpG binding protein 2
mhDISC1	Mutant human disrupted-in-schizophrenia 1
miRNA	Micro ribonucleic acid
NG2	Neural/glial antigen 2
NSAID	Nonsteroidal anti-inflammatory drug
O4	Oligodendrocyte marker
OPCs	Oligodendrocyte precursor cells
P	Postnatal day
PARP-1	Poly [ADP-ribose] polymerase 1
PDGF	Platelet-derived growth factor
PG	Prostaglandin
POA	Preoptic area
Poly:IC	Polyinosinic-polycytidylic acid
R	Receptor
SC	Schizophrenia
TLR	Toll-like receptor
TNF	Tumor necrosis factor
TSPO	Translocator protein
TX	Thromboxane
US	United States
